# The Emergence of Human Induced Pluripotent Stem Cell-Derived Cardiomyocytes (hiPSC-CMs) as a Platform to Model Arrhythmogenic Diseases

**DOI:** 10.3390/ijms21020657

**Published:** 2020-01-19

**Authors:** Marc Pourrier, David Fedida

**Affiliations:** 1 Department of Anesthesiology, Pharmacology and Therapeutics, University of British Columbia, Vancouver, BC V6T 1Z3, Canada; david.fedida@ubc.ca; 2 IonsGate Preclinical Services Inc., Vancouver, BC V6T 1Z3, Canada

**Keywords:** human induced pluripotent stem cell-derived cardiomyocytes, inherited cardiac diseases, arrhythmias

## Abstract

There is a need for improved in vitro models of inherited cardiac diseases to better understand basic cellular and molecular mechanisms and advance drug development. Most of these diseases are associated with arrhythmias, as a result of mutations in ion channel or ion channel-modulatory proteins. Thus far, the electrophysiological phenotype of these mutations has been typically studied using transgenic animal models and heterologous expression systems. Although they have played a major role in advancing the understanding of the pathophysiology of arrhythmogenesis, more physiological and predictive preclinical models are necessary to optimize the treatment strategy for individual patients. Human induced pluripotent stem cell-derived cardiomyocytes (hiPSC-CMs) have generated much interest as an alternative tool to model arrhythmogenic diseases. They provide a unique opportunity to recapitulate the native-like environment required for mutated proteins to reproduce the human cellular disease phenotype. However, it is also important to recognize the limitations of this technology, specifically their fetal electrophysiological phenotype, which differentiates them from adult human myocytes. In this review, we provide an overview of the major inherited arrhythmogenic cardiac diseases modeled using hiPSC-CMs and for which the cellular disease phenotype has been somewhat characterized.

## 1. Introduction

The unlimited source of human induced pluripotent stem cell-derived cardiomyocytes (hiPSC-CMs) provides new opportunities to create in vitro models of healthy and diseased human cardiac cells that can be used in drug safety and efficacy testing. Importantly, hiPSC-CMs also have the potential to become an essential tool to better understand the genetic basic of human cardiovascular diseases [1]. It is anticipated that major clinical applications will eventually include diagnosis and personalized treatment to predict therapeutic responses (beneficial or harmful) in individual patients with arrhythmogenic syndromes in vitro [2,3]. This approach, called “clinical trials in a dish” has been embraced by the Food and Drug Administration (FDA) in an effort to further develop and validate more predictive models to support drug development [4,5,6]. Limitations of animal models have generally hampered unravelling the underlying molecular and cellular mechanisms of cardiac disease, slowing development of new drugs. Historically, heterologous expression systems (e.g., HEK cells heterologously expressing a disease-specific mutation) and animal models (small and large) have played an important role in studying the pathophysiological mechanisms of arrhythmias and in developing targeted therapies [7,8]. Transgenic animal models have largely contributed to our current understanding of the pathogenic mechanisms associated with these diseases [7]. However, major differences in cardiac electrophysiological properties between small animals and humans largely limit the extrapolation of results, making the translation of results to humans difficult [8]. For example, the heart rate in mice is 8–10 times faster than that of humans, and ventricular repolarization is carried by potassium currents other than the delayed rectifier potassium channels (I_Kr_ and I_Ks_), the main two repolarizing currents in man [9,10]. Additionally, calcium handling differs in mice vs. human, resulting in a negative force-frequency relationship in mice [11]. Likewise, non-cardiac human cell lines (e.g., HEK-293 or CHO cells), are not good cardiac models since they do not express the native cardiac proteins required to reconstitute the complex cardiac structure and phenotype (e.g., sarcomere organization, calcium handling, metabolism, and electrophysiology).

Use of hiPSC-derived cardiomyocytes is broad, from in vitro applications (e.g., cardiotoxicity screening, drug discovery, disease modeling) to in vivo applications (e.g., cell replacement therapy).

In this review, we focus on the use of hiPSC-CMs for disease modeling. We first outline arrhythmogenic diseases modeled using hiPSC-CMs and describe the various experimental approaches used to investigate disease mechanisms and drug response.

## 2. The Human Cardiac Action Potential

The ventricular action potential profile is shaped by the orchestrated opening and closing of many ion channels, each with its unique time- and voltage-dependent amplitudes [10]. The rapid rate of depolarization (upstroke velocity) of the action potential (phase 0) results from a large inward current through voltage gated sodium channels (peak I_Na_). Peak I_Na_ determines the excitability of the myocardial tissue as well as fast conduction of the electrical impulse throughout the heart. Even though the AP lasts for 400 ms or more, peak I_Na_ in each myocyte lasts only 2–3 ms before the Na channels inactivate, which in combination with the activation of transient outward K currents (I_to_) result in a transient repolarization phase (phase 1). This early, rapid repolarization phase or “notch” controls the height and duration of the plateau phase (phase 2), which relies on the fine balance of inward (Ca and Na) and outward (K) currents. Although most Na channels are inactivated during the plateau phase, some Na channels continue to conduct, or even reactivate at membrane potentials encountered during the plateau (late I_Na_). During the plateau phase, opening of L-type Ca channels located in the t-tubules leads to the entry of Ca into the cells, which in turn activates the nearby ryanodine receptor (RyR2) within dyadic clefts, resulting in a large release of Ca from the sarcoplasmic reticulum (SR). This calcium induced calcium release (CICR) is central to excitation–contraction coupling [12]. I_Ca,L_ declines during phase 2 as the L-type Ca channels undergo Ca and voltage-dependent inactivation. The combination of Ca influx through L-type Ca channels and release from the SR raises the free intracellular Ca concentration, allowing Ca to turn on the contractile machinery [13]. As Ca channels inactivate, the outward K currents predominate, while the driving force for the K efflux through the voltage-gated K channels becomes high during the plateau. This results in the final repolarization phase (phase 3), where the membrane potential returns toward diastolic levels. The action potential duration is not only influenced by the height and duration of the plateau phase, but also by the time and voltage-dependent characteristics of the underlying voltage-gated Na, Ca, and K currents. In addition, the region-specific distribution of ion channel expression [14] generates various action potential morphologies in the heart to accommodate the physiological transmission of impulses from the sinoatrial node through the atria to the ventricles and the generation of a normal cardiac sinus rhythm in the heart [9,10]. Consequently, functional changes in any of these channels could have important consequences for the electrophysiological properties of the heart (i.e., action potential shape, refractory periods, and cardiac rhythms).

### 2.1. General Properties of Human Induced Pluripotent Stem Cell-Derived Cardiomyocytes (hiPSC-CMs)

#### 2.1.1. hiPSC Differentiation into Cardiomyocytes 

Human iPSCs provide the ability to capture the heterogeneity that develops from gender, ethnicity, and biological variability specific to the patients from which they have been derived [15]. Methods to generate hiPSC-CMs are reviewed extensively elsewhere [1,8,15,16,17]. Briefly, somatic cells are obtained mainly from the patient’s skin (dermal fibroblasts), blood (peripheral blood cells, i.e., T cells), urine (renal tubular cells), hair follicles (keratinocytes), fat and oral mucosa, and are reprogrammed to pluripotent stem cells using the method pioneered by Yamanaka and colleagues [18,19]. The resulting iPSCs are then differentiated into cardiomyocytes through exposure to a variety of stimuli [20,21,22] and culture media [22,23]. In most cases, around 90% of cells are cardiac troponin 2 (TNNT2)-positive [24,25]. Although hiPSC differentiation protocols usually generate a large proportion of ventricular-like hiPSC-CMs [23], a mixed population of ventricular-like, atrial-like, and sinoatrial node-like cells are typically produced [26,27,28,29]. In the context of disease modeling, it is essential to develop differentiation protocols that generate homogeneous populations of subtype specific cardiomyocytes to determine cell specific disease phenotypes. Current protocols effectively differentiate iPSCs toward the ventricular cell subtype [24,25,30]. However, more efficient and reproducible differentiation protocols to generate the atrial cell subtype have been described [25]. A protocol based on the activation of the retinoic acid signaling cascade to initiate atrial cardiomyocyte differentiation generates over 85% of the atrial subtype [25]. Differences in electrophysiological characteristics (i.e., action potential morphology and duration) and calcium handling (duration and kinetics of calcium transients) between ventricular-like, atrial-like, and nodal-like cardiomyocytes is key to the phenotypic analysis, confirming the subtype specific cell type.

#### 2.1.2. Electrophysiological Phenotype of hiPSC-CMs

**Figure 1:**
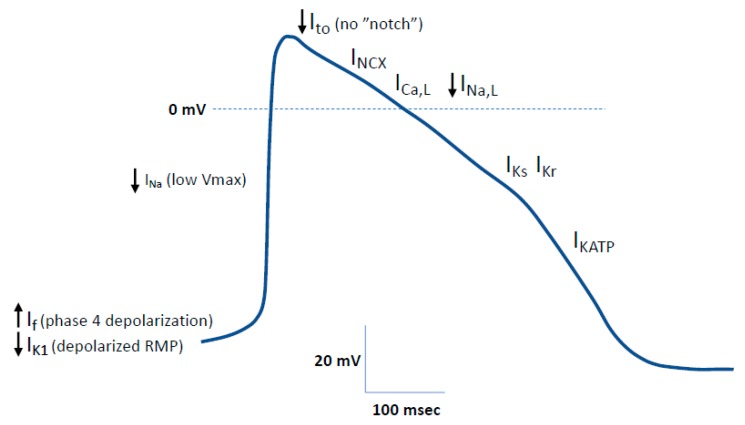
Typical action potential recorded from a ventricular-like human induced pluripotent stem cell-derived cardiomyocyte (hiPSC-CM) showing functional underlying ionic currents. Arrows indicate reported up and down regulation in comparison to adult human ventricular myocytes.

Human iPSC-CMs are generally considered electrophysiologically immature when compared to isolated adult human ventricular myocytes as reflected by a more positive maximum diastolic potential (MDP), a large phase 4 depolarization, a slower upstroke velocity, and almost absent notch [28,31,32]. The presence of a pacemaker current combined with a small I_K1_ in both ventricular and atrial-like hiPSC-CMs results in spontaneous beating cells, a characteristic of fetal human cardiomyocytes (Figure 1). Other ionic currents present in adult human cardiac myocytes have been identified in hiPSC-CMs [33]. Most importantly, individual ion channels in hiPSC-CMs functionally contribute to the action potential morphology with some ionic currents in hiPSC-CMs responding to adrenergic and cholinergic modulation (Figure 1) [28,31,33]. Despite a low upstroke velocity (Vmax) at the baseline resulting from reduced availability of Na channels and depolarized resting membrane potential, lidocaine reduces Vmax and decreases APD_50_ and APD_90_ at 1 Hz. This indicates that Na channels are functional as also supported by successful modeling of Na channel-based diseases (Table 1). Likewise, I_KATP_ activation with nicorandil shortens APD_50_ and APD_90_. In contrast, inhibition of I_to_, I_Kr_, and I_Ks_ with 4-AP, E-4031, and chromanol 293B, respectively, increases APD_50_ and APD_90_ [33]. Although the gene underlying I_KACh_ is expressed, activation of this current with carbachol did not affect APD, indicating its limited role in modulating AP in hiPSC-CMs [33]. In agreement with the depolarized RMP and the small I_K1_, barium (I_K1_ inhibitor) does not modulate APD [33]. The Ca-handling properties of hiPSC-CMs can also be described as immature as the lack of T-tubules result in poor coupling between the Ca entry through I_Ca,L_ and Ca release from the SR through RyR2 [34]. In addition, unlike adult human ventricular myocytes, which are characterized by a positive force frequency relationship (FFR) due to increased SR Ca load at higher frequencies [35], most differentiation protocols result in hiPSC-CMs with a neutral or negative FFR [36,37]. Similarly, rat and mouse myocytes often show negative FFR due to high SR Ca content, even at low frequencies [38].

Despite their differences in electrophysiological characteristics with adult human myocytes, hiPSC-CMs are generally considered as a predictive tool in the context of drug testing [39] and as a promising alternative to animal testing with regard to disease modeling (Table 1). 

Among the most broadly studied diseases using hiPSC-CMs based models are arrhythmic disorders caused by mutations in ion channel-related proteins, modulatory proteins, and structural proteins. 

### 2.2. Technical Considerations for the Electrophysiological Characterization of hiPSC-CMs

With the emergence of hiPSC-CMs, new tools are being used to study their electrophysiological profile, with multiple technologies available that offer costly but adequate throughput for screening applications including automated patch clamp and fluorescent imaging techniques to measure intracellular calcium concentration [40]. However, one of the limitations of fluorescence microscopy is the cytotoxicity of most dyes [9]. Impedance is used to monitor cell viability and contractility, and microelectrode array (MEA) is the only high-throughput platform compatible with multiwell plates that provides a predictive in vitro electrophysiological assay. The MEA system is an electrically-based technique that uses microelectrodes embedded at the surface of each well of a cell culture plate to measure changes in the electrical activity of the cells under the form of extracellular field potentials (FP) [41]. The FP signal is generated from spontaneously beating or electrically stimulated hiPSC-CMs. Disturbances to the recorded FP signal can be used to predict the identity of ion channel(s) targeted upon exposure to test compounds. In contrast to standard electrophysiology approaches such as patch clamp, the MEA technique is non-invasive since the electrical activity measured on each electrode is obtained without disrupting the cellular membrane. It is also label-free as dyes are not required. The recorded change in “extracellular field potential” is correlated with a body surface electrocardiogram (ECG). MEA systems accurately capture fast-acting events like cardiac depolarization (initial FP spike) as well as subtle T-wave changes for field potential duration calculation (FPD, a surrogate for QT interval), and evaluation of cardiac repolarization. Due to its non-invasive nature, MEA recordings can be taken over time on the same culture (hours-long continuous data collection or repeated reads of the same plate over days, hours, or months), an advantage that traditional techniques such as patch clamp cannot match. Additionally, each electrode on the microelectrode array is capable of recording or stimulating the overlying cell culture, thus allowing for the monitoring and control of cellular network behavior in each well. As spontaneous beating can be inconsistent between the cells and wells, it is essential to control it by either electrical stimulation or by optical pacing. The so called optogenetics approach consists of expressing the channelrhodopsin-2 in hiPSC-CMs, which upon activation by a light source triggers electrical activity [42,43]. Combined with the MEA platform, this optical approach allows artifact-free stimulation for pacing cardiomyocytes.

Others have reviewed the technologies available to perform functional analysis of hiPSC-CMs in more detail [9,43,44].

## 3. Inherited Arrhythmogenic Diseases

### 3.1. Congenital Long QT Syndrome (LQTS)

Long QT syndrome (LQTS) is an inherited cardiac disorder characterized by delayed ventricular repolarization as demonstrated by an abnormal QT interval prolongation on the surface ECG [45] with increased risk of developing a polymorphic ventricular tachycardia known as Torsades de Pointe (TdP) and sudden cardiac death [46]. LQTS occurs in more than 1 in 2000 people worldwide with a slight prevalence in females [47,48]. LQTS is currently associated with hundreds of mutations in 17 different genes encoding ion channels and ion channel-modulating proteins [48], but in more than 90% of all patients, it involves loss of function mutations in genes encoding KCNQ1 (LQTS1) and the human ether-a-go-go-related gene (hERG) (LQTS2) and gain of function mutations in Nav1.5 (LQTS3) [49,50]. At the cellular level, LQTS mutations result in the prolongation of the repolarization phase of the action potential, due to a decrease in net outward current secondary to an increase in I_Ca_, late I_Na_, and/or a decrease in I_Kr_, I_Ks_, or I_K1_ [51]. Critical action potential duration (APD) prolongation can lead to membrane potential oscillations during the plateau phase or later during repolarization, called early after depolarizations (EADs). In the presence of a suitable substrate, EADs constitute a trigger for developing TdP, which can degenerate into ventricular fibrillation [52]. Detailed description of the various types of LQTS are described in multiple comprehensive reviews [3,48,53,54,55,56,57,58,59,60,61]. More recently, a new clinical entity related to, but nevertheless distinct from LQTS, known as calmodulinopathy, has been described and studied in patient-derived hiPSC-CMs [62].

### 3.2. The Brugada Syndrome (BrS) 

Brugada syndrome (BrS) is an inherited disorder characterized by ST segment elevation (due to a prominent J wave) in the right precordial leads of the ECG, occurring either spontaneously or under sodium channel blocker challenge (e.g., ajmaline) [63]. BrS is associated with a risk of ventricular fibrillation and sudden cardiac death in young adults with structurally normal hearts [63,64]. Its prevalence is believed to range from 1 in 5000 to 1 in 2000, with a higher incidence in males [64]. Loss of function mutations in SCN5A, the gene encoding the Nav1.5 sodium channel account for 30% of all cases in which a gene variant is involved [64]. Nav1.5 dysfunction in the presence of a transmural voltage gradient caused by heterogeneous transmural distribution of the transient outward current (I_to_) results in an accentuation of the AP notch, and loss of spike and dome morphology, which leads to the development of phase 2 reentry and polymorphic VT [65,66]. Other mechanisms involve decreased conduction within the right ventricular outflow tract due to reduced I_Na_ [67]. Mutations in other ion channels involved in early repolarization have also been associated with BrS [68,69]. To date, 19 genes encoding either Na, K, or Ca channels have been associated with BrS [70].

### 3.3. Catecholaminergic Polymorphic Ventricular Tachycardia (CPVT)

Catecholaminergic polymorphic ventricular tachycardia (CPVT) is an arrhythmogenic disease characterized by syncope or cardiac arrest triggered by physical exercise or emotional stress [71]. It has a higher prevalence in males and typically occurs in individuals without cardiac abnormalities, with sudden cardiac death as the first symptom [48,72,73]. CPVT type 1 is caused by a gain of function mutations in the cardiac ryanodine receptor type 2 gene (RYR2), the ion channel responsible for releasing calcium from the SR into the cytosol. CPVT type 2 involves loss of function mutations in the gene encoding calsequestrin, an SR calcium buffering protein [74]. Those mutations result in a spontaneous calcium release (calcium leak) from the SR, which in turn provokes delayed after depolarizations (DADs), membrane potential oscillations that arise after full repolarization of the action potential. When DAD amplitude reaches the membrane threshold potential, a DAD-induced triggered action potential arises. [75]. In the presence of a suitable substrate, ventricular fibrillation can develop [76]. A loss of function mutation in the gene encoding RyR2 has also been identified [71]. Decrease in the peak of Ca release during systole, SR calcium overload, random bursts of calcium release, Na/Ca exchanger (NCX) activation, and early after depolarizations have been identified as the mechanisms triggering arrhythmias [71]. 

### 3.4. Short QT Syndrome (SQTS)

Short QT syndrome (SQTS) is a congenital disease characterized by very short QT interval on the ECG, syncope, palpitations, ventricular and atrial arrhythmias, and family history of sudden cardiac death [59,77]. SQTS is a rare disease, with a prevalence estimated to be less than 1 in 10,000 [59,78,79,80,81], but it could well be underdiagnosed because some affected individuals never experience symptoms [82]. There are eight identified variants involving gain of function mutations in genes encoding K channels (SQT1-3) or loss of function mutations in genes encoding Ca channels (SQT4-6), Na channels (SQT7), and the anion exchanger (SQT8) [83]. 

### 3.5. Arrhythmogenic Right Ventricular Cardiomyopathy (ARVC)

Arrhythmogenic right ventricular cardiomyopathy (ARVC) is an inherited disease characterized by progressive loss of myocardium, predisposing to a large spectrum of clinical presentations including ventricular tachycardia and ventricular fibrillation with an augmented risk of sudden cardiac death [84,85]. It is also named by the more general term arrhythmogenic cardiomyopathy (ACM), which also recognizes the involvement of the left ventricle [85,86]. ACM develops during adolescence and young adulthood with a prevalence between 1 in 5000 and 1 in 1000 depending on the population [85,87]. Plakophilin 2 (PKP2), a desmosomal protein, is the most commonly mutated gene [85]. However, other desmosomal proteins such as desmoplakin (DSP) and desmoglein 2 (DSG 2) are also involved [85]. Rare mutations have been reported in proteins of the adherens junction, cytoskeletal structure, ion transport, and cytokines [85]. These pathogenic mutations result in cardiomyocyte loss, fibrosis, adipogenesis, inflammation, and arrhythmogenesis [85].

### 3.6. Hypertrophy Cardiomyopathy (HCM) 

Hypertrophy cardiomyopathy (HCM) is the most common cardiac inherited disease, with a prevalence of 1 in 500 to 1 in 200 in the general population of healthy young adults worldwide [88,89,90]. HCM is characterized by left ventricular wall thickening (hypertrophy), hypercontractility, impaired relaxation (diastolic dysfunction), increased energy consumption, and sudden cardiac death in the absence of any other cardiovascular conditions such as hypertension and valvular diseases [91,92]. Although more than 2000 different variants have been identified in at least 11 sarcomere- and myofilament-associated genes [93], the most prevalent pathogenic mutations (nearly 50% of all cases) are found in two genes encoding sarcomeric proteins: MYH7, encoding the β-myosin heavy chain, and MYBPC3, encoding the cardiac myosin-binding protein C (cMyBP-C) [91]. However, more recently, the hypothesis that disease heterogeneity could not be solely explained by a single sarcomeric protein variant has been proposed [93]. Pathological features of HCM include mechanical and electrical disturbances, which lead to diastolic dysfunction and ventricular arrhythmias. While increased myofilament calcium sensitization contributes to the development of hypertrophy and diastolic dysfunction, hypertrophy-induced remodeling of cardiac ion channels likely contributes to the worsening of diastolic dysfunction and serves as a substrate for the development of fatal arrhythmias [88,89,94,95].

### 3.7. Dilated Cardiomyopathy (DCM) 

Inherited dilated cardiomyopathy (DCM) is characterized by left ventricular or biventricular dilation and the reduction of mechanical force generation (i.e., systolic dysfunction) in the absence of hypertension, valvular, congenital, or ischemic heart disease [91,96,97]. Pathogenic mutations are reported in over 50 genes encoding proteins of the sarcomere, nuclear membrane, cytoskeleton, outer cellular membrane, extracellular matrix, ion channels, mitochondria, and splice-regulating proteins. Together, these mutations account for about 30% of all DCM cases [91]. The prevalence of DCM is uncommon in children (from 1 to puberty), being highest in the first year of life and much less common afterward. In adults, the prevalence is estimated to be 1 in 250/500 [90]. Arrhythmogenic factors in DCM include structural abnormalities such as fibrosis and scarring, which contribute to abnormalities in conduction and refractoriness, thereby providing a substrate for reentrant arrhythmias [98]. At the cellular level, decreased I_to_ and I_K1_ most likely contribute to the action potential prolongation. 

Although it is undeniable that heterologous expressions systems and animal models have allowed us to better understand cellular and molecular mechanisms associated with these conditions, limited access to human cardiomyocytes and the incapacity to model patient-specific diseases with currently available preclinical models has significantly restrained the study of these diseases.

## 4. Human Induced Pluripotent Stem Cell-Derived Cardiomyocytes (hiPSC-CMs) as a Model to Study Inherited Arrhythmogenic Cardiac Diseases

**Figure 2:**
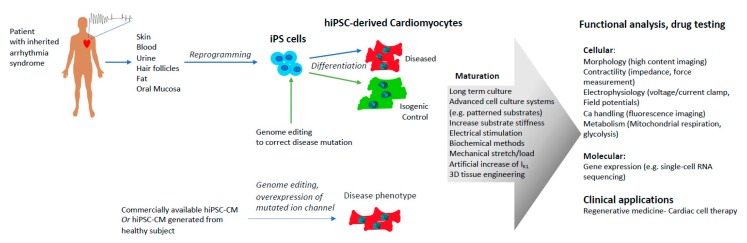
Current approaches to model arrhythmogenic diseases using human induced pluripotent stem cell-derived cardiomyocytes (hiPSC-CMs). Cells can go through a maturation process before functional readout.

Disease modeling possibly represents the most valuable use of hiPSC-CMs. Currently, hiPSC-CM technology has been used to model a number of inherited cardiac diseases, whether by generating patient-specific cells, editing the genome of healthy cells, or overexpressing mutated ion channels (Figure 2).

### 4.1. Patient-Specific hiPSC-CMs

Production of pluripotent stem cells from human adult somatic tissues provides the opportunity to generate large numbers of patient-specific stem cells, which makes it by far the most reported method to model arrhythmogenic diseases (Table 1). As such, hiPSC-CM represents a critical preclinical model system for investigating the genetic basis of human cardiovascular diseases. An advantage over animal models is that hiPSC-CM is closely genetically matched to patients with a particular disease.

Among the most broadly studied diseases using hiPSC-CMs based models are arrhythmic disorders caused by mutations in ion channel-related proteins, modulatory proteins, and structural proteins. LQTS is the most common channelopathy and numerous studies have been published on LQTS patient-derived hiPSC-CMs (Table 1). Functional characterization is mostly obtained via patch clamp electrophysiological techniques and micro electrode arrays (MEA) where APD or FPD prolongation are used to measure delayed repolarization and after depolarization-type arrhythmias. In 2010, Moretti et al. became the first group to model LQTS using hiPSC-CMs [99]. They reported the screening of a family affected by LQTS type 1 and identified a mutation in KCNQ1, the gene encoding the ion channel carrying the slow delayed rectifier potassium current I_Ks_. They generated functional iPSC-CM, which maintained the disease genotype and phenotype of LQTS1 (i.e., decreased I_Ks_, APD prolongation, and increased susceptibility to catecholamine-induced tachyarrhythmias [99], Table 1), which recapitulate the stress-induced clinical syndrome [48]. Others have generated LQTS1 patient-specific hiPSC-CMs and showed a similar phenotype (decreased I_Ks_, increased APD, increased sensitivity to APD prolonging drugs (e.g., decrease in repolarization reserve)) (Table 1). In fact, KCNQ1 mutations affecting both trafficking and gating mechanisms of the channel were successfully modeled using this approach [99,100]. Mutations in the KCNH2 gene, which encodes the pore-forming channel subunit (hERG) conducting the rapid delayed rectifier potassium current, I_Kr_, are linked to LQTS2 [101]. The phenotypes of LQTS2 patient-specific hiPSC-CMs include decreased I_Kr_, APD prolongation, EADs, and increased drug sensitivity (Table 1). A large proportion of KCNH2 mutations are associated with hERG trafficking defects. Using hiPSC-CMs generated from LQT2 patients, Mehta et al. showed that lumacaftor, a clinically used drug acting as a chaperone during protein folding, was able to reverse the LQT2 phenotype [102]. LQTS3, which results from a gain of function mutation of the Nav1.5 channel has also been modeled using patient specific hiPSC-CMs (Table 1). As expected, characterization of LQTS3 cells showed alteration of I_Na_ biophysical properties leading to increased late I_Na_, with some frequency dependence with regard to drug response. APD/FPD prolongation and EADs were also observed [103,104,105,106,107]. Na channel inhibition with mexiletine reversed the phenotype [108]. In contrast, Brugada syndrome is characterized by a loss of function mutation in Nav1.5 and sometimes overlaps with LQTS3 to generate a mixed phenotype [109]. Davis et al. generated iPSC lines containing an SCN5A mutation, causing a cardiac Na channel LQT3/BrS overlap syndrome [104]. They successfully recapitulated the mixed phenotype, highlighting the utility of hiPSC-CMs in modeling more complex cardiac diseases. However, in a more recent study, Okata et al. failed to recapitulate the mixed LQT3/BrS phenotype of the SCN5A E1784K mutation in hiPSC-CMs [110]. In those cells, only the LQTS3 phenotype could be reproduced (increased late I_Na_, APD/FPD prolongation). Interestingly, knocking down the expression of SCN3B, a modulatory subunit expressed at low levels in adults, unmasked the BrS phenotype. Other, less common LQT syndromes have been studied in patient specific hiPSC-CMs such as LQTS7 (Andersen-Tawil syndrome), which is characterized by a loss of function mutation in Kir2.1 channels (conducting I_K1_) [111], and LQTS8 (also known as Timothy Syndrome), which is associated with a loss of function mutation in the Cav1.2 subunit (conducting I_Ca,L_). hiPSC-CMs generated from LQTS8 patients are characterized by irregular contraction, altered Ca handling, increased APD, and irregular electrical activity [112]. Of the LQTS that are not associated with mutations in gene encoding ion channels, LQTS14 and LQTS15 (associated with mutations in the gene encoding calmodulin 1 and 2, respectively) are the only ones that have been modeled using patient specific hiPSC-CMs [3,113,114,115]. LQTS-associated CaM mutations, also called calmodulinopathies, are known to alter the Ca/CaM binding affinity [116], and involve multiple potential targets such as I_Ca,L_ [115]. As a result, hiPSC-CMs generated from LQTS14 and LQTS15 patients are characterized by increased I_Ca,L_ and delayed repolarization [113,114,115].

Several groups have generated hiPSC-CMs from CPVT1 and CPVT2 patients [117,118,119,120,121,122,123] (Table 1), where most reported altered Ca handling and catecholamine-induced DADs, a hallmark of the disease.

Disease modeling in HCM has been limited by the lack of appropriate animal models. For example, most HCM mutations are found in the MYH7 gene, which is the main β-myosin heavy chain isoform in the human heart, but not in small animals such as rodents. This is a major limitation that can now be addressed using HCM patient-specific hiPSC-CMs, which have been shown to recapitulate the human phenotype: impaired relaxation, increased myofilament Ca sensitivity, APD prolongation, and larger L-type Ca current [124]. In the clinic, and in contrast to HCM, dilated cardiomyopathy (DCM) is characterized by ventricular dilatation and left ventricular systolic dysfunction, which can result in progressive heart failure and arrhythmias [125]. Consistent with the clinical phenotype, hiPSC-CMs isolated from the DCM patient generally show contraction and Ca handling abnormalities (Table 1).

Electrophysiological findings of less commonly studied patient-specific hiPSC-CMs from Duchenne muscular dystrophy, Friedreich’s ataxia, Barth syndrome, and familial atrial fibrillation are also reported in Table 1. Overall, despite differences between hiPSC-CMs and adult human cardiomyocytes with regard to their structural and electrophysiological characteristics, these studies demonstrate the utility of patient-specific hiPSC-CMs to model arrhythmogenic conditions. However, before engaging into phenotyping studies, the expression profile of known key protein targets may be required in some cases to ensure that the molecular environment recapitulates the pathophysiology of the disease [110]. 

In addition, it is important to acknowledge that using patient-specific hiPSC-CMs for disease modeling has limitations. Although differentiation protocols are more efficient, generating iPSCs from patients remains time-consuming and somewhat unpredictable. A major limitation of this approach is the lack of isogenic control. In early studies, control iPSCs were generated from fibroblasts isolated from healthy donors. [29,99]. Although “similar” to the patient specific iPSC lines, such controls are not reflective of the patient genetic background and epigenetic modifications induced by epidemiological and environmental factors. With the emergence of genome editing techniques, the pathophysiological phenotype can now be reversed to generate the proper isogenic control.

### 4.2. Genome Editing

Rapidly advancing genome editing tools including clustered regularly interspaced short palindromic repeats (CRISPR/Cas9), zinc finger nucleases (ZFN), adenoviral vectors, and transcription activator-like effector nucleases (TALEN) can significantly reduce the time and effort it takes to generate human cell lines carrying specific gene mutations or other variants with rare off-target mutagenesis [151,152,153,154,155,156,157]. Cellular genome editing has already established itself as a powerful tool and as an alternative to animal models to investigate pathophysiological mechanisms in various cardiac diseases [129,136,155,156,158]. 

Introducing desired mutations into existing (commercially or through academic collaborations) hiPSC-CM lines has several advantages over generating hiPSC-CM lines from patients: it is quicker and less expensive than recruiting and obtaining consent from patients, collecting somatic cells, reprogramming, performing quality control, and engaging into differentiation protocols [1]. In some cases, patients carrying a rare variant of interest might not be available for recruitment, making genome editing a method of choice.

Genome editing provides the unique opportunity to study disease modifying variants in the same genetic background. In contrast to patient-specific hiPSC, this potentially reduces the influence of epigenetic modifications and other genetic modifiers such as environmental factors on the pathophysiological phenotype [1,158]. For example, Wang et al. used the zinc finger nuclease (ZFN) technology to introduce KCNQ1 and KCNH2 dominant negative mutations in hiPSC-CMs generated from healthy donors [130]. This approach had no impact on the whole genome expression profile and was successful in recapitulating LQTS phenotypes (APD prolongation and EADs) [130]. The same pathological phenotype was observed in hiPSC-CMs generated from a patient carrying the same LQTS mutation, thus validating the genome editing approach. This technique is also amenable to drug testing [130]. In studies using patient-specific hiPSC-CMs, genome editing can be integrated in order to correct the known variant and create a more appropriate control (i.e., isogenic control) for comparison [113,133,138,150] (Table 1). In those cases, targeted gene correction can reverse the pathological phenotype and normalize APD values. Gene editing in the context of reversing a pathological phenotype has been applied in multiple arrhythmogenic conditions including LQTS [113], SQTS [137], BrS [138], HCM [143], and Barth syndrome [150].

Despite recent improvements in gene editing, which generally limit off target effects, there are other limitations that are specific to the differentiation protocols such as a lack of consistency, mixed population of cardiomyocytes rather than specific cell subtype, and immaturity of the differentiated cells.

### 4.3. Overexpression of Mutated Genes in hiPSC-CMs

Standard transfection methods have also been used to genetically perturb commercially available hiPSC-CMs. Gelinas et al. used commercially available cells as a means to screen a novel KCNJ2 variant. Using this approach, the authors showed that Kir2.1-52V is associated with a prolongation of the action potential duration with some evidence of arrhythmic activity [135]. 

Similarly, fusogenic liposomes can be used as carriers for the transport of mRNA load into the cytoplasm of a large variety of mammalian cells including hiPSC-CMs. This method has the advantage of using very short incubation times, which allows the gentle transfer of mRNA into the host cell. This method is highly efficient in non-proliferating cells such as neurons and cardiomyocytes. The LQTS2 was modeled in commercially available cells using this approach [134]. The dominant negative KCNH2 mutation (G628S) was transfected in Cor.4U cells. In G628S transfected cells, RMP and APA were unchanged, whereas APD_50_ and APD_90_ were slightly increased. G628S cells were more sensitive to dofetilide than the control cells. In dofetilide, APD_90_ and APD_50_ were increased by 71% and 23%, respectively. In four out of eight cells, EADs were recorded. These results suggest that overexpression of dominant negative mutations of ion channels in hiPSC-CMs might be used as a model of cellular arrhythmogenic diseases (e.g., LQT syndrome) to evaluate the proarrhythmic liability or efficacy of test molecules. 

## 5. Limitations of Using Human Induced Pluripotent Stem Cell-Derived Cardiomyocytes (hiPSC-CMs) to Model Inherited Arrhythmogenic Cardiac Diseases

A major debate remains whether or not, fetal-like cells derived from iPSCs with immature structure and function as compared to human adult cardiac myocytes can appropriately reproduce the molecular modification and the clinical phenotype in an adult disease [92].

Despite the enormous advancement of the iPSC-CM field in the last few years, this technology is still not generally accepted as an alternative to current animal models as the lack of maturity of the cardiomyocytes’ action potential morphology is still a major limitation [159,160]. Although most ionic currents found in adult ventricular myocytes are also present in hiPSC-CMs (Figure 1), the electrophysiological phenotype of hiPSC-CMs is generally characterized as being immature, which is reflected by spontaneously beating cells resulting from a less negative resting membrane potential (reduced I_K1_) and the presence of the pacemaker current (I_f_). The more positive resting membrane potential explains the slower upstroke velocity (Vmax). In addition, structural and functional properties of mitochondria in hiPSC-CMs are also consistent with an immature phenotype [161,162]. With regard to metabolism, in contrast to adult cardiomyocytes that depend on fatty oxidation as a source of energy, hiPSC-CMs mostly rely on the glycolytic pathways for energy production [163] Contractile proteins are highly disorganized, as indicated by random structures of the myofibrils [164], and lack of T-tubules [165].

Despite these limitations, hiPSC-CMs have been validated in multiple studies, where they have consistently recapitulated the clinical phenotypes observed in the donors and demonstrated the expected phenotypes associated with drug effects (Table 1) [39]. 

Some level of maturity can be reached by enhancing the function or expression level of I_K1_ [166,167]. I_K1_ not only maintains the resting membrane potential close to the equilibrium potential for potassium, but it also contributes to phase 3 repolarization. The advantages of I_K1_-enhanced iPSC-CM include the absence of spontaneous beating (and therefore allow the possibility of controlling electrical pacing), and a resting membrane potential close to physiological values (i.e., −80 mV) [167]. Other strategies to improve the maturation of hiPSC-CMs include long term culture [168] and specific biochemical methods such as using the hormone triiodothyronine [169] or fatty acids [170]. The bioengineering approach, which consists of generating engineered three-dimensional human cardiac tissue, and applying electrical stimulation or mechanical strain to improve both structural and functional maturation, shows great promise [171]. A more detailed discussion on strategies to improve maturation of hiPSC-CMs is beyond the scope of this review, but other reviews have treated this subject extensively [160,172,173,174].

As alluded to before, another limitation is that typically, the hiPSC-CM population is a mix of atrial, ventricular, and nodal cells. This mixed population can have significant implications for downstream modeling of disease states or drug screening [22]. However, improving the ability to derive specific cardiac subtypes from pluripotent sources has been the focus of recent work [24,25,27,175,176,177].

Overall, maturation of hiPSC-CMs remains a significant hurdle to the wider application of these cells to preclinical and, eventually clinical studies for the application of regenerative medicine. In this context, low survival rate, immunogenicity, tumor formation, and arrhythmia also limit their potential clinical applications [178].

## 6. Conclusions

Strategies to achieve functional and structural maturation as well as the production of specific cardiac subtypes will enable more comprehensive studies of disease modeling and will widen the potential applications of hiPSC-CMs in preclinical studies. New technologies including human organoids and engineered human tissue mimicking the 3D structure and function of native cardiac tissue appear to be viable alternatives to currently used cell and animal models [161,162,179,180,181,182,183,184]. Native-like in vitro environments obtained from mixing various cell populations and suitable biomaterials may provide the means to generate a mature electrophysiological phenotype, currently lacking in 2D cultures [161,185]. 3D bioprinting currently allows the rapid fabrication of living human tissue with cardiac-like patterns using different primary cell types [186]. 3D bioprinted human cardiac tissues are expected to improve the predictive accuracy of the non-clinical drug discovery process by providing industry and researchers with the technology to create highly customized physiologically relevant 3D human tissue models (healthy and diseased) for developing new therapeutic options [187]. This technology has the potential to drive a fundamental shift in the pharmaceutical industry, resulting in the development of entirely new therapeutics, and enabling pharma to test drugs shelved due to a lack of appropriate models. Notably, these novel human-based assay technologies are expected to significantly reduce laboratory animal use, supporting an industry-wide mandate to reduce, refine, and replace animal testing. Clearly, engineered human cardiac tissue represents an exciting new avenue for drug testing and disease modeling.

## Figures and Tables

**Table 1:** Selection of reports using human induced pluripotent stem cell-derived cardiomyocytes (hiPSC-CMs) to model arrhythmogenic diseases.

Disease Modeled	Gene Mutation	Main Findings	Approach	Ref
LQTS1	KCNQ1 R190Q	Decreased I_Ks_ due to trafficking defect, altered channel activation and deactivation, APD prolongation, increased susceptibility to catecholamine-induced tachyarrhythmias, attenuated by treatment with beta blockade.	Patient-specific hiPSC-CMs	[99]
LQTS1	KCNQ1 1893delC	Decreased I_Ks_ due to trafficking defect, increased cFPD, E4031-induced EADs and polymorphic ventricular tachycardia-like arrhythmias, insensitivity to chromanol 293B.	Patient-specific hiPSC-CMs	[100]
LQTS1	KCNQ1 308~344del	Decreased I_Ks_, APD prolongation reversed by ML277.	Patient-specific hiPSC-CMs	[126]
LQTS1	KCNQ1 G269S	Increased APD_50,70,90_. High incidence of arrhythmias including EADs. Increased sensitivity to cardiotoxicity caused by hERG blockade.	Patient-specific hiPSC-CMs	[127]
LQTS1	KCNQ1 c.1022C>T	Reduced chromanol 293B-sensitive I_Ks_, Increased APD	Patient-specific hiPSC-CMs	[128]
LQTS1	KCNQ1 c.478-2A>T c.1781G>A	APD and FPD prolongation and decreased or no I_Ks_	Patient-specific hiPSC-CMs and CRISPR/Cas9	[129]
LQTS1 LQTS2	KCNQ1 G269S KCNQ1 R190Q KCNQ1 G345E KCNH2 A614V	Increased APD, EADs Pharmacological characterization with nifedipine, pinacidil	Zinc finger nuclease (ZFN)-mediated targeted gene addition into AAVS in iPSCs and patient specific iPSC	[130]
LQTS2	KCNH2 A614V	APD prolongation, reduction of I_Kr_, EADs and triggered arrhythmias. Increased cFPD. Evaluation of potency of pharmacological agents on disease phenotype.	Patient-specific hiPSC-CMs	[29]
LQTS2	KCNH2 G1681A	APD and FPD prolongation. E4031 and isoprenaline-induced EADs. AP shortening in the presence of potassium channel openers and in some cases, abolition of EADs.	Patient-specific hiPSC-CMs	[131]
LQTS2	KCNH2 R176W	APD prolongation, reduced I_Kr_. Increased sensitivity to sotalol and E4031, more pronounced inverse correlation between the beating rate and repolarization time compared with control cells.	Patient-specific hiPSC-CMs	[127]
LQTS2	KCNH2 A561P	Decreased I_Kr_ due to hERG trafficking defects, APD prolongation, higher incidence of EADs and increased sensitivity to E4031.	Patient-specific hiPSC-CMs	[132]
LQTS2	KCNH2 A614V N996I	Decreased I_Kr_, Prolongation of FPD, increased sensitivity to E4031 (hERG inhibitor). Targeted gene correction rescued the wild type phenotype.	Patient-specific hiPSC-CMs.	[133]
LQTS2	KCNH2 G628S	Increased APD_90_, increased sensitivity to dofetilide	Overexpression of mutated ion channel in Cor.4U cells	[134]
LQTS2	KCNH2 S428X R366X A561V IVS9-28A/G	Increased FPDc, Ca-handling defects and proarrhythmic events at 2 Hz. Lumacaftor restored trafficking in trafficking deficient mutants.	Patient-specific hiPSC-CMs	[102]
LQT3	SCN5A ΔKPQ	Faster recovery from Na channel inactivation, increased late I_Na_, prolonged APDs and EADs at low pacing rates.	hiPSC-CMs from a mouse model	[103]
LQT3, BrS	SCN5A 1795insD	Decreased peak I_Na_, smaller Vmax and longer APD_90_, increased late I_Na_.	Patient-specific hiPSC-CMs	[104]
LQTS3	SCN5A V1763M	Increased APD, increased TTX-sensitive late I_Na_, positive shift of steady state inactivation and faster recovery from inactivation. Mexiletine reversed the LQT3 phenotype.	Patient-specific hiPSC-CMs	[108]
LQTS3	SCN5A F1473C	Increased late I_Na_. Positive shift of steady state inactivation, faster recovery from inactivation. Pronounced rate dependence of I_Na_: reduced late I_Na_ and increased late I_Na_ block by mexiletine with increasing pacing rate.	Patient-specific hiPSC-CMs	[106]
LQTS3	SCN5A V240M SCN5A R535Q	Increased APD_50_ and APD_90_, longer time to peak and longer time to 90% inactivation in I_Na_ recordings.	Patient-specific hiPSC-CMs	[107]
LQTS3, BrS	SCN5A E1784K	Increased cFPD and APD_90_ (ventricular-type cells). Increased in late I_Na_, no change in peak I_Na_. LQTS3/BrS iPSC-CM recapitulate the LQT3 phenotype, not the BrS phenotype. Knockdown of SCN3B in LQTS3/BrS showed decreased peak I_Na_, negative shift of the steady state inactivation, thus unmasking the BrS phenotype in LQTS3/BrS iPSCs.	Patient-specific hiPSC-CMs; siRNA used for knockdown	[110]
LQTS7 (ATS)	KCNJ2 R218W	Higher incidence of irregular Ca release, strong arrhythmic events, reversed by flecainide through the modulation of I_NCX_.	Patient-specific hiPSC-CMs	[111]
LQTS7	KCNJ2 G52V	Native I_K1_ induced by electrical pacing resulted in more negative RMP. G52V abolished native I_K1_, depolarized RMP, decreased cell excitability. In transfected cells that generated an AP: Increased APD, arrhythmic activity (EADs and spontaneous activity).	Transient transfection of Cor.4U hiPSC with TransIT-LT1	[135]
LQTS8 (Timothy syndrome)	CACNA1C G406R	Irregular contraction, excess calcium influx, increased APD, irregular electrical activity and abnormal calcium transients in ventricular-like cells	Patient-specific hiPSC-CMs	[112]
LQTS8	CACNA1C N639T	Increase in ERP (in optically stimulated cells), increased APD, slower voltage-dependent inactivation of Cav1.2.	CRISPR/Cas9	[136]
LQTS15	CALM2-N98S	Lower beating rates, increased APD, impaired inactivation of L-type Ca channels. Specific ablation of the mutant allele using CRISPR-Cas9 rescued the electrophysiological abnormalities of LQTS15-hiPSC-CMs.	Patient-specific hiPSC-CMs, CRISPR/Cas9	[113]
LQTS15	CALM1 F142L	Increased I_Ca,L_ due to severe impairment of I_Ca,L_ Ca-dependent inactivation, Prolonged FPD and APD, failure of repolarization to adapt to high rates. Verapamil reversed the mutation-induced repolarization abnormalities.	Patient-specific hiPSC-CMs	[114]
Short QTS	KCNH2 T618I	Increased I_Kr_ (current density and enhanced membrane expression). Shorter APD, increased beat to beat variability. I_Kr_ inhibition restored a normal phenotype.	Patient-specific hiPSC-CMs, CRISPR/Cas9	[137]
BrS	SCN5A R620H and R811H SCN5A 4189delT	Decreased I_Na_ (lower membrane expression), abnormal AP profiles, closely coupled single trigger beat, sustained triggered activity, reduced Vmax, Ca transient abnormalities.	Patient-specific hiPSC-CMs, CRISPR/Cas9	[138]
CPVT	RyR2 F2483I	Catecholaminergic stimulation-induced DADs, higher amplitudes and longer durations of spontaneous Ca release events at basal state. CICR events continued after repolarization and were abolished by increased cytosolic cAMP levels.	Patient-specific hiPSC-CMs	[117]
CPVT	RYR2 M4109R	Higher incidence of DADs, increased frequency and magnitude of after-depolarizations in the presence of isoproterenol and forskolin, triggered activity. Flecainide and Thapsigargin eliminated DADs. Whole cell Ca transient irregularities worsened with adrenergic stimulation and Ca overload, improved with beta blockers. Store-overload-induced Ca release.	Patient-specific hiPSC-CMs	[118]
CPVT	RYR2 S406L	Increased diastolic Ca concentrations, reduced sarcoplasmic reticulum Ca content, increased susceptibility to DADs and arrhythmia in the presence of catecholaminergic stress. Increased frequency and duration of elementary Ca sparks. Dantrolene restored normal Ca spark phenotype and reversed the arrhythmogenic phenotype.	Patient-specific hiPSC-CMs	[119]
CPVT	CASQ2 D307H	Isoproterenol-induced DADs, oscillatory arrhythmic prepotentials, after contractions and elevation of diastolic Ca concentrations. CPVT iPSC-CMs had a more immature phenotype than control cells (less organized myofibrils, enlarged sarcoplasmic reticulum cisternae and reduced number of caveolae.	Patient-specific hiPSC-CMs	[120]
CPVT	RYR2 E2311D	DADs in resting state and in the presence of isoproterenol. Non-homogeneous spreading of calcium transients (aggravated with isoproterenol). KN-93, a CaMKII inhibitor reversed the arrhythmic phenotype.	Patient-specific hiPSC-CMs	[121]
CPVT	RyR2 F2483I	Aberrant unitary calcium signaling, smaller calcium stores, higher CICR gains, and sensitized adrenergic regulation.	Patient-specific hiPSC-CMs	[122]
CPVT	RyR2 P2328S	Increased non-alternating variability of Ca transients in response to isoproterenol. Epinephrine decreased AP V_max_.	Patient-specific hiPSC-CMs	[123]
ARVD/C	PKP2 c.2484C>T	Exaggerated lipogenesis and apoptosis, calcium handling deficits (prolonged Ca transient relaxation), corrected by introducing the wild type PKP2 gene back into mutant iPSC-CMs.	Patient-specific hiPSC-CMs	[139]
ARVD/C	PKP2 c.972InsT/N	Prolonged field potential rise time, widened and distorted desmosomes. Clusters of lipid droplets were identified in ARVC iPSCs with the most severe desmosomal pathology. Exposure of the cells to apidogenic stimuli augmented desmosomal distortion and lipid accumulation.	Patient-specific hiPSC-CMs	[140]
ARVC	PKP2 c.1841T>C	Reduced cell surface localization of desmosomal proteins, adipogenic phenotype.	Patient-specific hiPSC-CMs	[105]
HCM	MYH7 R663H	Dysregulation of Ca cycling, elevation of intracellular Ca concentration, cellular enlargement, arrhythmias (DADs).	Patient-specific hiPSC-CMs	[141]
HCM	MYH7 R663H	High incidence of arrhythmias, including DADs. Increased sensitivity to cardiotoxicity caused by hERG blockade.	Patient-specific hiPSC-CMs	[127]
HCM	SCO2 E140K SCO2 G193S	Ultrastructural abnormalities, diminished response to isoproterenol and caffeine, DADs, increased beat rate variability	Patient-specific hiPSC-CMs	[142]
HCM, WPW	PRKAG2 R302Q	Abnormal firing patterns, DADs, triggered arrhythmias, increased beat to beat variability. CRISPR correction reversed the mutation phenotype.	Patient-specific hiPSC-CMs CRISPR/Cas9	[143]
HCM	T247M	Impaired relaxation, Increased myofilament Ca sensitivity, APD prolongation, Increased I_Ca,L_	Patient-specific hiPSC-CMs	[124]
DCM	TNNT2 R173W	Altered Ca handling, decreased contractility, abnormal sarcomeric α-actinin distribution, isoproterenol-induced reduced beating rates, compromised contraction, and more cells with abnormal sarcomeric α-actinin distribution.	Patient-specific hiPSC-CMs	[144]
DCM	TNNT2 R173W	Very low frequencies of irregular electrophysiological waveforms. Increased sensitivity to APD_90_ shortening by nicorandil.	Patient-specific hiPSC-CMs	[127]
DCM	DES A285V	Decreased maximum rate of Ca ion reuptake, slower spontaneous beating rate, diminished response to isoproterenol.	Patient-specific hiPSC-CMs	[145]
DCM	PLN R14del in	Ca handling abnormalities, electrical instability, abnormal cytoplasmic distribution of PLN protein and increases expression of molecular markers of cardiac hypertrophy in iPSC-CMs.	Patient-specific hiPSC-CMs	[146]
DCM	TTN c.8607dupA TTN c.70690dupAT	Decreased response to isoproterenol, elevated external Ca concentration and Angiotensin-II. Altered response to caffeine.	Patient-specific hiPSC-CMs	[125]
DMD	DMD c.5899C>T	Slower spontaneous firing rates, decreased If, DADs, prolonged APD, increased I_Ca,L_	Patient-specific hiPSC-CMs	[147]
Familial AF	Multiple genetic variants	Higher beating rate, increased I_Ca,L_ and I_f_. Prolonged APD, no changes in Ca handling. Stress-induced DADs.	Patient-specific hiPSC-CMs.	[148]
Friedreich’s ataxia	FXN Expanded GAA repeat mutation	Increased beating rate variability, calcium handling defect as implied by decrease Ca transients.	Patient-specific hiPSC-CMs	[149]
Barth syndrome	TAZ c.517delG TAZ c.328T>C	Sparse and irregular sarcomeres, contraction abnormalities	Patient-specific hiPSC-CMs CRISPR/Cas9	[150]

LQTS, Long QT Syndrome; APD, Action Potential Duration; EAD, Early After Depolarization; APD_50,70,90_, Action Potential Duration at 50%, 70%, 90% repolarization; FPD, Field Potential Duration; hERG, human Ether-a-go-go-Related Gene; AAV, Adeno-Associated Virus; cFPD, Field Potential Duration rate corrected; BrS, Brugada Syndrome; ATS, Andersen-Tawil Syndrome; RMP, Resting Membrane Potential; ERP, Effective Refractory Period; CRISPR, Clustered Regularly Interspaced Short Palindromic Repeats; CPVT, Catecholaminergic Polymorphic Ventricular Tachycardia; RyR2, Ryanodine Receptor 2; CaMKII, Ca-Calmodulin dependent protein Kinase II; DAD, Delayed After Depolarization; CICR, Ca-induced Ca release; ARVD/C, Arrhythmogenic Right Ventricular Dysplasia/Cardiomyopathy; HCM, Hypertrophic Cardiomyopathy, WPW, Wolff-Parkinson-White Syndrome; DCM, Dilated Cardiomyopathy; PLN, Phospholamban; DMD, Duchenne Muscular Dystrophy; AF, Atrial Fibrillation.
